# From medical officers of health to multidisciplinary public health specialists: a history of the professional transformation of public health in Britain, 1970–2025

**DOI:** 10.1017/mdh.2025.10039

**Published:** 2026-01

**Authors:** David Evans

**Affiliations:** Emeritus Professor, Centre for Public Health and Wellbeing, University of the West of England, Bristol

**Keywords:** Multidisciplinary, Non-medical, Profession, Professional project, Public health, Specialty

## Abstract

Throughout the twentieth century, senior roles in UK public health were reserved for doctors. Local authority medical officers of health were replaced in 1974 by NHS community physicians and from 1989 by medical directors of public health. Over the last decade of the century, an increasingly vocal group of non-medical public health professionals sought to break the glass ceiling that restricted them from advancing to senior roles; although they received encouragement from some leaders within the Faculty of Public Health Medicine, there was also significant resistance from many members. A number of factors came together around the year 2000, which culminated in a ground-breaking decision by the English Department of Health to allow non-medical appointments as directors of public health and consultants in public health in the NHS, with the then Secretary of State memorably declaring it was time to ‘take public health out of the ghetto’. At the same time, the leadership of the Faculty of Public Health Medicine overcame opposition from some of its members and opened its training, examinations, and membership to non-medical candidates. By the early 2020s, half of the renamed Faculty of Public Health members were from backgrounds other than medicine as well as 90% of directors of public health in England. This paper explores the complex history behind this unprecedented opening of a medical specialty to non-medical membership, the factors that enabled it, and the continuing legacy of tensions and inequalities within an occupation that is both a medical specialty and a multidisciplinary profession.

## Introduction

In 2021, 90% of the local authority directors of public health (DsPH) in England came from backgrounds other than medicine.^1^ This situation would have been unthinkable for the first three quarters of the twentieth century, when a medical qualification was an essential requirement for local authority medical officers of health (MOsH). As late as 2001, all the National Health Service (NHS) DsPH were medical, although by that date the government had clearly indicated that non-medical DsPH were soon to be appointed. This opening of senior roles in a medical specialty to candidates from other disciplinary backgrounds is virtually unique in the history of the medical profession in the UK and deserves further study and exploration.^2^ The abolition of the MOH role in 1974 has been well documented by social scientists and historians, not least by Jane Lewis in her classic book *What Price Community Medicine*, as well as in a series of articles and chapters in the late 1980s and early 1990s.^3^ Lewis argues that MOsH had lost their way in the mid-century and particularly following the advent of the NHS in 1948. Lewis recounts what she sees as a series of failures – of social medicine to capture the hearts of service (i.e. non-academic) public health doctors in the 1940s, and of public health doctors limiting themselves to the role of medical administrators. Underlying these weaknesses, she sees a continuing identity crisis, driven by a central paradox: ‘if public health is about the health of the people, then much more is involved than medicine ….Yet any such widening of public health’s focus threatens to weaken an already weak specialty further.’^4^ Other historians, particularly Martin Gorsky, have taken a more positive view of the achievements of MOsH in the period before the role was abolished.^5^

Whilst views differ on the achievements of MOsH prior to abolition in 1974, there is consensus amongst both the profession and historians that the transition to community physicians in the NHS was largely unsuccessful, and that for a time public health medicine lost its way. This has been well documented in a witness seminar and a number of other accounts, including an official review of the public health function by the then Chief Medical Officer for England, Donald Acheson, in 1988.^6^ Closely linked to the abolition of the MOH and the transition to the community physician was the establishment of the Faculty of Community Medicine (FCM). This key moment of professional development has also been extensively documented by the profession and by historians.^7^ One of several heated debates at the time was the question of whether the new Faculty should be exclusively medical or whether senior non-medical academics represented by the Society for Social Medicine should be included. Ultimately, the decision was taken to limit membership to those with a medical qualification. Importantly, there was no discussion of admitting any non-medical service professionals nor any concept at the time of the public health profession being a multidisciplinary endeavour.^8^

The story of the movement in the 1990s to open senior public health posts to candidates from backgrounds other than medicine has been told a number of times, including first-hand accounts in two witness seminars, and in a special issue of the journal *Public Health.* ^9^ Jenny Wright and colleagues have written the most detailed account, including a number of previous unpublished personal communications from significant players, particularly for the key period of change in the late 1990s and early 2000s.^10^ Most of these narratives have been more descriptive than theoretical, but one contemporary account applied Freidson and Larson’s concept of the professional project drawn from the study of clinical medicine to make sense of the medical resistance to change.^11^

The professional project describes the way that occupational groups (and in particular medicine) have sought to establish their professional status through training, qualifications, registration, and regulation. Within health care, medicine is widely seen as the most successful professional project having used its claims to expert knowledge to subordinate other occupational groups including nurses. There is no one historiographical overview of other UK health occupations seeking to professionalise, although there are useful introductions to the issues in texts by Susan Nancarrow and Alan Borthwick, and Gerry Larkin.^12^ Accounts exist for the emergence and development of several UK health occupations, for example, radiographers, paramedics, and genetic counsellors.^13^ The most successful professionalising occupations were those like physiotherapists, radiographers, and dietitians which were recognised as professions supplementary to medicine by the government in 1960 and went on to have protected titles, recognised academic qualifications, professional societies, and state registration.^14^ There is not, however, another example of a UK occupational group seeking to establish equal status with medicine or to be recognised as equivalently expert with doctors in a medical specialty.

Applying the concept of the professional project to public health, public health doctors in the twentieth century were largely autonomous in controlling the technical content of their work and dominant in the division of public health labour. Their technical expertise was largely equated with epidemiology, biostatistics, and communicable disease control. Public health doctors had a monopoly on senior local authority posts (to 1974) and subsequently NHS positions, successfully excluding other occupational groups through the mechanisms of exclusive medical recruitment, exacting training requirements, and (from 1972) Faculty membership.^15^

Many of the accounts of the rise of multidisciplinary public health were written in the early to mid-2000s when the key changes had only recently taken place, and most do not take account of the longer historical perspective back to the 1970s, nor include critical recent developments such as the transfer of the public health function back from the NHS to local authorities in England in 2013.^16^ Finally, most of these accounts focus narrowly on professionals employed specifically as public health specialists and do not take account of important wider contextual factors, in particular the rise and fracturing of the health promotion professional project which fed into multidisciplinary public health.^17^

Overall, there is a need to bring together and synthesise the mass of material – first-hand accounts by public health service professionals, contemporary accounts by public health academics and social scientists and historians’ accounts (which generally only cover the first part of the period prior to the 1990s). This paper sets out to provide a comprehensive account of the transition from medical public health leaders in the period 1970s–1990s to multidisciplinary public health specialists taking leadership roles from the 2000s to 2020s. It explores the complex history behind this unprecedented opening of a medical specialty to non-medical membership, the factors that enabled it, and the continuing legacy of tensions and inequalities within an occupation that is both a medical specialty and a multidisciplinary profession. Critically, this paper applies the theory of the professional project to this history, adding a theoretical perspective that is missing from most existing accounts. The paper concludes with three key arguments. First, that in the late 1990s, a combination of pressure from government and non-medical colleagues obliged public health doctors to adapt and expand their professional project to include multidisciplinary professionals within the scope of specialist public health. Second and relatedly, there have been continuing efforts to ensure that multidisciplinary public health remains enmeshed in a largely unquestioned web of medically dominated structures (i.e. training, examinations, registration, and regulation) designed to maintain a firm boundary between elite public health specialists and the larger group of non-elite public health practitioners. Third, it has proved increasingly challenging for the leaders of the profession to manage the tension of being both a medical specialty and a multidisciplinary profession.

## Prelude to change – the profession of public health in 1970

In 1970, the profession of public health was generally viewed as synonymous with medical doctors specialising in public health. The vast majority of public health doctors were employed by local authorities as MOsH or as their deputy or assistant MOsH. Public health doctors were distinguished from other medical specialties by having passed the Diploma in Public Health examination, which the General Medical Council (GMC) recognised as the appropriate qualification for entry on the medical register as a public health specialist.^18^ Other members of MOH departments included social workers, sanitary inspectors, health visitors, and health education officers, but they were regarded as different occupations and not normally considered as part of the profession of public health. The Diploma examination was offered by the London School of Hygiene and Tropical Medicine, the Royal Institute of Public Health and Hygiene, and several other universities. By 1970, the examination’s reputation was generally poor, widely regarded as insufficiently rigorous and too traditional in content.^19^ It was, however, a statutory requirement for appointment as a MOH. Public health doctors were similarly held in generally low esteem by their clinical colleagues, particularly by consultants in higher status hospitals.^20^ MOsH were represented professionally by the Society of MOsH and occupationally by the British Medical Association Public Health Committee.

Academically, there were two fairly distinct types of public health departments. A small number of departments of social medicine including Oxford, Edinburgh, and Birmingham were research active and generally concerned with the wider determinants of population health; several provincial medical schools had appointed their local MOH as professor of public health but due to their heavy service responsibilities, they were often not very research active.^21^ They were also likely to operate relatively small Diploma in Public Health programmes and focus on service public health issues. Academic public health was represented by the Society for Social Medicine, founded in 1956, and which included both medical and non-medical academics active in research. Many commentators at the time and latterly considered there to have been an unhealthy divide between academic and service public health.^22^

Two key occupational groups within the MOH’s empire were social workers and sanitary inspectors (later environmental health officers), both of which were seeking to professionalise and to gain their independence from the MOH. Beginning with the Seebohm Committee, a series of major inquiries and legislative changes in the late 1960s and early 1970s led to the establishment of separate social services and environmental health departments within local authorities.^23^ A third major occupational group, health visitors, community nurses, and midwives, was transferred into the NHS but no longer managed by or directly linked to public health doctors in their new role as community physicians. The smaller and more disparate group of health education officers also transferred into the NHS and often continued to be managed by public health doctors. Health education officers are particularly significant for our story as in their later manifestation as health promotion officers they were a significant contingent within the movement for multidisciplinary public health in the 1990s. But in 1970, they were far from professionalised, with a Society of Health Education Officers, but no specific educational qualification, standardised training, or professional registration. Indeed, of the other occupations within the MOH’s empire, only health visitors and midwives were professionally registered (with the General Nursing Council or the Central Midwives Board), registrations which were of course deemed much inferior to medical registration.

## End of the MOH era, the public health profession in transition, 1970–1974

As well as the pressures for change already mentioned coming from the Seebohm Committee and planned reforms of local government, the profession faced pressure for change from within the medical establishment and indeed, from within the specialty of public health medicine itself. The story has been told in detail by Michael Warren, but is worth briefly summarising here.^24^ There was a widespread anticipation of the need for change within the MOH world,^25^ more widely in local government and the NHS and in the medical establishment. A key catalyst was the 1968 report of the Royal Commission on Medical Education (the Todd Report).^26^ The report recommended that ‘In community medicine there is a great need for a professional body which can bring together all the interests, academic and service, and which has the support and strength to undertake the assessment needed during and at the end of general professional training.’^27^ Following a complex series of discussions detailed by Warren, a proposal was drafted for a Faculty of the three Royal Colleges of Physicians of London, Edinburgh, and Glasgow. The new Faculty was to bring together all the major public health bodies of the day, with the crucial exclusion of the non-medical members of the Society for Social Medicine. Medical members of the Society had argued strongly for the Faculty to include non-medical public health academics but other public health doctors feared this would risk the approval of the new Faculty by the prestigious medical Royal Colleges. Ultimately, the lure of association with the Royal Colleges was too strong for most of the service public health doctors and membership was limited to those medically registered. To address this disappointment, the final proposal included the statement: ‘At a later date, by agreement with the Royal Colleges, consideration would be given to the admission to membership of non-medical colleagues practising, teaching or researching in the field of community medicine.’^28^ Notably, however, the actions of the founders of the Faculty in excluding non-medical members were wholly consistent with Freidson and Larson’s theories of the exclusionary characteristics of the professional project.

The proposal for the creation of the Faculty was accepted by the Royal Colleges, a Provisional Council was held in 1971 and applications for foundation membership were invited. The Faculty was formally inaugurated in 1972. Early professionalising tasks included devising the content and procedures for the examination of membership of the Faculty, and developing a programme of specialist training acceptable to the Joint Committee on Higher Medical Training and the Council for Postgraduate Medical Education. The first membership examinations took place in November 1974 and the examination replaced the Diploma in Public Health as the key qualification for public health specialist practice; the previous statutory requirement for MOsH to hold the Diploma became redundant with the abolition of the MOH role and the advent of the NHS community physician in the same year. Community physicians were appointed at all three levels of the reorganised NHS: as regional medical officers in regional health authorities, area medical officers in area health authorities, and community physicians in district management teams.^29^

A further and related key professional development was the inauguration of new Master’s courses, variously titled Social Medicine, Community Medicine, or Public Health, first at the London School of Hygiene and Tropical Medicine in 1972 and subsequently in other universities. Attendance at such courses became an expected part of the preparation for the Faculty membership examination and the specialist training programme, emphasising the expert knowledge that set public health doctors apart from other public health workers. These Master’s courses were initially limited exclusively to doctors pursuing public health specialist training but notably some institutions, and in particular the London School, also started to provide postgraduate courses at Diploma or Master’s level in Health Education in this period.

## Community medicine in the wilderness and rise of health promotion, 1974–1988

By all accounts, the move of public health doctors into the NHS as community physicians in 1974 was detrimental to the profession.^30^ There were some positives. For the first time, public health doctors were on the same salary scales and terms and conditions as hospital consultants. The establishment of the Faculty raised the status of the specialty, and public health trainees now followed similar registrar/senior registrar pathways to clinical trainees. Notionally, community physicians had important roles to play in planning, managing, coordinating, and evaluating health services. The reality for most community physicians was quite different. There were numerous problems. In practice, community physicians’ roles and responsibilities were not clear. They lacked the support teams that public health doctors were used to in MOH days. They were outnumbered by hospital clinicians, lacked clear authority, and were not even allowed to be called ‘consultants’, thus suggesting they were lower down in the implicit hierarchy of medical specialties.^31^ The NHS went through a further series of reorganisations, in particular the 1982 abolition of the area health authority level and the impact of the Griffiths review of NHS management.^32^ After 1982, district health authority areas were mainly not coterminous with local authorities, thus losing the important link to a defined population. The number of community physicians declined with early retirements and problems with recruitment to the specialty. Multiple accounts speak of low morale and pessimism about future career prospects during this period.^33^

Despite the initial stated intention for the Faculty ‘at a later date’ to consider the membership of non-medical colleagues, this does not appear to have occurred in the period before the 1990s. The one extant mention of any early discussion within community medicine comes from a report of a study group on *Rethinking Community Medicine*, which suggested the creation of a non-medical ‘Community Health Adviser’ role of equivalent status to community physicians.^34^ This report referenced a recent Faculty workshop where one of the main sessions addressed the question: ‘Do community physicians need to be doctors?’ The authors commented: ‘Whilst most of the doctors present unsurprisingly seemed to think that they did, the argument that some ten years or so of extremely expensive training could better be used to produce two non-medical specialists found at least some support from the conference.’^35^ There is no evidence, however, that either the workshop session or the study group report led to any formal discussion of the issue by the Faculty Board during this period.

At the same time that community medicine appeared to be in decline, health education (later evolving into health promotion) was on the rise. The first significant inquiry into the role of health education officers was the Cohen Report of 1964, which led among other things to the setting up of the Health Education Council in England and the Scottish Health Education Unit (later Group).^36^ Both of these bodies were to play important roles in the subsequent professional development of the health education officer role. With the 1974 NHS reorganisation, a circular was issued spelling out for the first time how health education was to be organised in the NHS. Health education officers were placed at the area level, managed by area medical officers. Probably only around 200 transferred to the NHS in 1974 but over the next decade their numbers steadily increased, and then jumped in the mid-1980s with the advent of ring-fenced HIV/AIDS funding for prevention. By the early 1990s, numbers probably reached around 1000.^37^ Crucially, the Health Education Council was committed to the professional development of health education officers and supported the creation of a number of new postgraduate Diploma and Master’s programmes in the 1980s; from just two in 1974, these increased to 27 by the early 1990s. The growth of health education/promotion teams and academic units was supported by wider developments in the international health promotion movement including the Canadian Lalonde Report, the WHO’s *Ottawa Charter for Health Promotion*, *Health for All by the Year 2000*, and *Healthy Cities* initiatives.^38^ Significantly, there was a large degree of overlap between this international health promotion movement and what some community physicians were promoting as ‘the new public health’, also concerned with health inequalities and the wider social determinants of health.^39^

## The Acheson Report 1988 and the revival of public health medicine

Following outbreaks of communicable diseases at two NHS hospitals, and an unusually high number of deaths, the Secretary of State for Social Services asked the Chief Medical Officer for England, Donald Acheson, to chair a committee to investigate the outbreaks and consider the future development of community medicine.^40^ The report was quite blunt in portraying the variation in the quality and impact of community medicine. Whilst some community physicians ‘created vigorous departments which continue to make important contributions to the planning and development of health services’, others ‘simply failed to make the transition’, contributing to the ‘failure of the specialty to establish its professional standing’.^41^ Acheson made a number of recommendations to revitalise the NHS public health function including renaming the specialty as public health medicine, requiring the appointment of a medical DPH in every health district with a minimum complement of consultants in public health medicine, and re-establishing annual reports on the health of the population. In addition, there were several recommendations for improving the training of public health specialty trainees.

The report acknowledged the multidisciplinary nature of public health and called for multidisciplinary training to be more available. Specifically, the reports recommended ‘that the relevant training institutions and professional bodies should discuss how best to achieve multi-disciplinary awareness and collaboration in the training of public health practitioners, including the possibility of establishing a school or schools of public health’.^42^

There was, however, no detailed discussion of what form such multidisciplinary training might take, as there was for the substantial further discussion of proposed changes to public health medicine training. No mention was made of the extensive multidisciplinary training already being provided by university Master’s programmes in Health Promotion. Neither was there any consideration for whether specialty training or Faculty examinations and membership might be opened to non-medical candidates. Whilst acknowledging that some countries, for example the USA, had leadership roles for non-medical specialists, the report explicitly argued that in the UK, public health specialists needed to be doctors.^43^ It claimed that public health doctors ‘unlike his/her predecessor, cannot expect to sit as of right at the head of a large hierarchy. He or she is but one member of a team of specialists in various aspects of public health’.^44^ How this supposed non-hierarchical working was to be squared with NHS management structures with DsPH and consultants managing more junior non-medical public health staff was not discussed.

Many of the Acheson report recommendations were implemented, in particular the appointment of DsPH in health authorities and an increase in the numbers of specialty registrars. Community physicians were renamed consultants in public health medicine and subsequently, the Faculty similarly changed its name to the Faculty of Public Health Medicine. Many commentators then and subsequently have seen the Acheson Report as a key moment in the revival of the profession of public health medicine.^45^ The one glaring omission was any action on the recommendation for relevant training institutions and professional bodies, the GMC, the NHS Training Authority, the regional health authorities, and medical schools to discuss how best to take forward multidisciplinary public health education and training.

## Increasing agitation for multidisciplinary public health, 1990s

In 1989, the then Conservative government announced the introduction of the NHS internal market, to be implemented by 1991.^46^ Health authorities were split between purchasers and providers of health services, which predictably resulted in a series of local reorganisations as hospital and community health services provider units were separated from purchasing health authorities. Some health authorities were then merged to create larger and notionally more effective purchasers. Inevitably, the purchaser-provider split had a huge impact on both public health doctors and on health promotion teams. Public health doctors usually remained in purchasing health authorities. As with previous reorganisations, a number took the opportunity to take early retirement. Health promotion teams were often moved into provider units, thus weakening any links between the two occupational groups. Some larger health promotion units also began to have multiple levels of seniority, with some senior health promotion officers gaining substantial management experience. A few health promotion team managers moved into purchasing health authorities, thus developing commissioning expertise alongside public health doctors. There was also often movement between service and academic health promotion units, thus some health promotion officers developed significant research expertise and/or doctoral degrees. The health promotion field was extremely multidisciplinary, with entrants drawn from nursing, teaching, social work, and wider social sciences. Thus, a critical mass began to form of a group of professionals who saw themselves as having some overlapping knowledge, skills, and competencies comparable to those of public health doctors and who were not afraid to critique the ‘medical model’ of public health. Finally, there was a substantial portion of the health education/promotion workforce who sought to professionalise, by developing their professional organisation, the Society of Health Education and Health Promotion Specialists, developing standards of qualification and a code of practice, and seeking professional registration.^47^ Thus there was for a time a parallel professional project for health education/promotion officers, claiming related but distinct forms of expert public health knowledge and skills.

At the same time, there was renewed pressure on the Faculty to address the continuing exclusion of non-medical colleagues from its training, examinations, and membership. A particularly forceful critic was Klim McPherson, a non-medical Professor of Public Health who recounts how he continually ‘ranted’ and ‘harassed’ various presidents of the Faculty about how crazy it was that he was teaching public health doctors, asked to be Scientific Adviser to the Faculty, yet could not be a member.^48^ The Faculty’s response to this and other pressures was to create the category of ‘Honorary Members’ in 1991, which was a very partial solution. As a later president of the Faculty reflected, honorary members ‘were neither honorary, because they had to pay a subscription, nor were they members, because they didn’t have any of the other rights of the members’.^49^ Not only did honorary members not have the right to vote or stand for office within the Faculty, but the status did not make them eligible for NHS DPH or consultant posts, so was much more relevant for academic than service public health professionals. But the creation of honorary members did establish the first formal breach of the boundary separating public health as a medical profession from other supposedly less expert occupations in public health.

Wider changes in the professional landscape also contributed to the growth of multidisciplinary public health, in particular the opening up of Master’s in Public Health courses to non-medical students, first in Cardiff in 1990, then the prestigious London School in 1992, and increasingly other Master’s programmes as well. This meant that individuals from other occupational groups could claim expert knowledge in public health equivalent to that held by public health doctors.

Thus, the agitation for non-medical membership of the Faculty and the opening of career opportunities for those from backgrounds other than medicine in senior service public health posts continued and broadened. Although it is impossible to point to one moment of origin, it was at this time in the early 1990s when a distinct multidisciplinary public health professional project can be seen to more consistently challenge the exclusionary boundaries set by public health medicine. A discussion document was issued to Faculty members in 1993 but ‘it elicited a wide range of views from which no consensus emerged’.^50^ A working group was set up in 1994, which commissioned a survey of non-medical professionals working in public health on their roles, relationships, and training needs. Although challenged by a lack of any database of such professionals or clarity on who might be relevant to approach, the authors successfully contacted and received usable replies from almost 1,000 individuals. The results demonstrated a wide diversity of roles and responsibilities, as well as academic and professional qualifications. Over 700 were qualified to first degree level, 500 to Master’s, and 195 had PhDs, including 60 working in service public health.^51^ Although there was great diversity in roles and responsibilities, two consistent messages were that most respondents had received little support for training and the vast majority desired more training.^52^

The results of the survey were presented at the first national conference for multidisciplinary public health in Birmingham in 1995, which ultimately led to the formation of the Multidisciplinary Public Health Forum. The Forum was not intended to be a long-term organisation; rather, it was a time-limited network of regional groups advocating for the opening of training and career opportunities.^53^ The work of the Forum was spurred on by a vote taken by Faculty members in 1996 where a majority voted against opening the Part 1 Examination to disciplines other than medicine. This left the then president and members of the Faculty executive who supported opening training and examination in a difficult position, as they had not been able to take a majority of their members with them, still less the seventy-five per cent then necessary to carry a motion at the Faculty annual general meeting.^54^ Although there were a number of statements in the medical press at the time seeking to argue why only public health doctors could fulfil a leadership role in public health, these claims to unique expertise were increasingly challenged by others who had both the technical competence and leadership experience in other settings. As several participants in the two later witness seminars testified, the real reasons were much more to do with medical protectionism, the fear that non-medical specialists would price doctors out of senior public health roles, and a general crisis of confidence within public health medicine.^55^

The Multidisciplinary Public Health Forum was keen to ensure that all public health professionals were rigorously trained, accredited, and regulated, thus accepting key tenets of the public health medicine professional project. In pursuing these objectives, the Forum was aided by the relatively lack of success of a parallel attempt to professionalise health promotion. For a number of years, the Society of Health Education and Health Promotion Specialists had been seeking to establish statutory training, registration, and regulation of health promotion specialists. But there was significant dissent within the ranks of the occupation from those who saw such professionalisation as an inappropriate medical model, excessively hierarchical, and likely to exclude the marginalised.^56^ The register was published in 1991 but only on a voluntary not a statutory basis, with no employer sign-up and with only 500 or so of the estimated 1,000 plus health promotion specialists joining.^57^ The Department of Health made it clear in 1996 that it would not support the health promotion professionalisation objectives.^58^ What it did mean, however, was that there was a substantial body of health promotion professionals, often in senior or managerial positions, who had accepted the arguments for rigorous training and professional registration and regulation, and often saw themselves as part of the public health workforce. Health promotion professionals proved a significant portion of those surveyed by Somervaille and Griffiths and brought extensive experience working in or alongside NHS public health doctors to align with the academics like McPherson who were already making the case within the Faculty but often without service experience.^59^

## Public health ‘out of the ghetto’, 1997–2003

The election of the New Labour government in 1997 radically changed the context in which discussions around multidisciplinary public health were being held. The new government gave a much higher priority to public health and tackling inequalities in health than its Conservative predecessor, and was also more sceptical of professional protectionism in the NHS. The Multidisciplinary Public Health Forum and its allies in the leadership of the Faculty took the opportunity to push forward with developing the framework for the education, development, and accreditation of multidisciplinary public health specialists. A ‘Tripartite Group’ was formed by the Forum, the Faculty, and the Royal Institute of Public Health, which was soon joined by observers from the four UK national health departments. Crucially, the English Chief Medical Officer Kenneth Calman^60^ was supportive and funding was made available by the NHS Executive to support the next key piece of work on *Feasibility Study of the Case for National Standards for Specialist Practice in Public Health.* ^61^ This work reflected both the aspirations of Forum members to be seen as equally concerned with high professional standards as medical public health specialists, but equally sought to reassure the Faculty that professional standards would be maintained if non-medical specialists were accepted. This professionalising strategy and the changing political climate bore its first important fruit with the publication of the emerging findings from Calman’s project to review the public health function. It explicitly called for different disciplines within public health to play ‘a full part, up to and including the most senior levels’ and stated a need to develop career pathways, accreditation systems, and equal opportunities for ‘public health specialists from a variety of backgrounds’.^62^ Despite continuing vocal opposition from some members of the Faculty, the constellation of factors favouring change was now overwhelming. With continued support from the new English Chief Medical Officer, Liam Donaldson^63^ and at least some regional DsPH, pressure from honorary members, the Multidisciplinary Public Health Forum, and the Tripartite Group, the Faculty Executive put the issue to the Faculty members once more in 1998 and this time they voted to open the Part 1 Examination and Diplomate membership to disciplines other than medicine.^64^ This was a, or perhaps the, crucial point of transition from a narrowly public health medicine profession project to a wider multidisciplinary public health professional project.

At the same meeting, the Faculty membership did not support opening up full membership to non-medical colleagues,^65^ but this was now virtually bound to follow eventually, not least because the increasing number of non-medical Diplomate members would now be eligible to participate in future votes. In fact, further change followed quite rapidly. In July 1999, the English public health white paper surprised many by committing to the creation of non-medical specialists in public health ‘which will be of equivalent status in independent practice to medically qualified consultants in public health medicine and allow them to become directors of public health’.^66^ In the same year, the first regional public health training schemes for non-medical candidates were introduced, although initially only in some regions and varying widely in pay, terms and conditions, and length of contract (between two and five years), but otherwise seeking to mirror the existing medical training scheme including first completing a Master’s in Public Health and then sitting the Part 1 Examination.^67^ There were inevitably some teething issues in setting up the schemes, including funding arrangements and some public health doctors’ initial reluctance to supervise non-medical trainees.^68^ By 2005, multidisciplinary schemes were operating in all regions in England and in Wales, although Scotland did not open its public health specialty training to multidisciplinary candidates until 2013 and Northern Ireland not until 2021.

Despite the clear evidence from the White Paper that ministers as well as the English Chief Medical Officer were supporting multidisciplinary public health, some public health doctors continued to put up a rear-guard resistance to change.^69^ The pages of the *British Medical Journal* and other medical publications saw a continued stream of articles and letters claiming only doctors could or should lead in public health.^70^ Possibly in response to such vocal opposition, the then Secretary of State for Health, Alan Milburn, gave a highly publicised speech in 2000:

[T]he time has come to take public health out of the ghetto. For too long the overarching label ‘public health’ has served to bundle together functions and occupations in a way that actually marginalizes them … [B]y a series of definitional sleights of hand the argument runs that the health of the population should be mainly improved by population-level health promotion and prevention, which in turn is best delivered—or at least overseen and managed—by medical consultants in public health. The time has come to abandon this lazy thinking and occupational protectionism.^71^

There could not have been a clearer expression of ministerial support for the changes, and though a minority of public health doctors continued to argue the case, health authorities soon followed by advertising consultant-level posts for non-medical specialists in public health. The following year, English health authorities were abolished and replaced by 303 primary care trusts which ministers announced could appoint non-medical DsPH. This was followed by the Faculty voting at its annual meeting for the Part 2 Examination to be opened and for full membership for disciplines other than medicine. As DsPH for the new English primary care trusts were appointed in 2002, the first 38 non-medical DPH appointments were made.^72^

Concerns shared by both the Multidisciplinary Public Health Forum and the Faculty to ensure quality-assured professional standards and accreditation for non-medical specialists led to intensive work to develop a registration and regulation process to parallel that provided for public health doctors by the GMC. Discussions began within the Tripartite Group but it was not until the Department of Health had announced the imminent appointment of non-medical consultants in public health and DsPH that the work took on a sense of urgency. By 2003, the then Minister for Public Health was able to officially launch the UK Voluntary Register for Public Health Specialists, later known as the UK Public Health Register (UKPHR).^73^ The register was voluntary as unlike the GMC it had no statutory basis, but it was essentially mandatory for anyone applying for a DPH or consultant in public health post, as registration would become an essential criterion for such posts, and the Faculty would ensure this was adhered to by employers in its role in all Advisory Appointments Committees.^74^ The establishment of the register was the critical final step in the multidisciplinary public health project being accepted (with varying degrees of enthusiasm or resignation) by public health doctors as it ensured that non-medical specialists adhered to similar (if not identical) training, accreditation, and regulation, thus maintaining a clear boundary between expert specialists and other occupational groups in public health.

A final symbolically important change in 2003 saw the Faculty vote to change its name to the Faculty of Public Health, by dropping the word Medicine from its title signalling the radical change from a medical to a multidisciplinary Faculty that had occurred over the preceding six years.

## What’s in a name? Generalist specialists, defined specialists, and non-specialist specialists, 2003–2020

The speed of change meant that a number of crucial decisions around professional processes needed to be taken in a very short period of time, around 2002-2003. Non-medical DsPH and consultants had been appointed in English health authorities before the voluntary register had been established, and so they were initially without professional registration or regulation. An early decision was to grant them two years provisional registration with the requirement that they submit a portfolio within two years to demonstrate they met the required professional standards. Unlike with the creation of the Faculty in 1972, no grandparenting without assessment would be allowed in order to reassure the field that appointments would maintain elite professional knowledge and skills; all other existing senior non-medical public health professionals would also need to submit a portfolio to gain acceptance on the register. Initially, this portfolio route was only intended to be open for three years with the intention that once the regional public health training schemes were open to non-doctors, there would no longer be a need for the portfolio route except for rare exceptional circumstances. Although this meant that some 400 specialists were registered by UKPHR by 2006, there were still many other non-medical professionals including some at a senior level with specialist expertise in particular areas (e.g. public health intelligence or health promotion) who were not able to meet the criteria for portfolio registration and for whom the training scheme was not appropriate. In 2006, the UKPHR took the decision to open a second route to registration by portfolio assessment for ‘defined specialists’, a decision which was again opposed by many within public health medicine as defined specialists were thought not to have demonstrated expertise and experience across all the domains of public health practice; it was not until 2013 that the Faculty offered defined specialists membership, whilst fellowships were routinely offered only to the so-called generalist specialists who had demonstrated competency across all the domains of public health either through the training programme or by portfolio.^75^ This distinction was not resolved until much later in 2017 when the UKPHR closed the defined specialist route and reintroduced a generalist specialist registration by portfolio route.^76^ By this point, several defined specialists had been appointed to DPH roles and it was generally recognised that the distinction was irrelevant in practice, and the Faculty quietly dropped resistance to offering fellowships to defined specialists.

A final element of ambiguity was an unanticipated consequence of the decision of the UK government to transfer the local public health function from the NHS to English local authorities in 2013.^77^ Local authorities were diverse in the roles and structures they developed for their public health teams, with the only statutory constraint being that they had to appoint a DPH. Many of them also had one or more consultants in public health transferred in, as well as a number of other public health staff. These more junior public health staff were given a number of different job titles, but in some cases these included ‘principal public health specialist’, ‘senior public health specialist’, and/or ‘public health specialist’, but with none of these posts requiring the postholder to be on the GMC or UKPHR specialist register.^78^ In a few cases, the job specifications for these posts even listed UKPHR practitioner registration as a criterion, a lower level of registration introduced by the UKPHR in 2011. Importantly, even the most senior of these postholders could not apply for consultant in public health posts, as local authorities continued to adhere to Faculty guidance that consultant posts required specialist registration.

## The birth, life, and precarious existence of UKPHR, 2003–2020

The Voluntary Register was established with significant support from the public health establishment as crucial to the professional project whatever doubts were harboured by some Faculty members. Successive ministers of public health supported it, the first chair of the Register was a past president of the Faculty, Jim McEwen, and the former Chief Medical Officer for England, Sir Kenneth Calman, chaired its initial Advisory Group. The English Department of Health agreed to fund the set-up costs of the Register in 2002 and provided ongoing funding for several years. Despite the funding, however, much of the effort that went into the Register was voluntary; the board and the portfolio assessors were all volunteers, and in the early days, the only paid employee was the registration administrator. Over time, UKPHR grew into an organisation with a small staff, including a professional chief executive. But in the period up to 2020, its staff never numbered more than five and at times were as low as two; it was of course dwarfed in size and influence by the GMC as the other main regulator of public health specialists. At times, its existence was precarious, both because its income was essentially limited to registration fees once the Department ceased direct financial support in the mid-2010s, and because the UK government was continually reconsidering its wider position on the regulation of health professionals in general, and sometimes on public health specialists in particular. There was also considerable pressure from some public health doctors for the GMC to take over the regulation of non-medical public health specialists, something that the GMC itself did not appear keen on. In 2010, the Department of Health published the results of a review it had commissioned from the regional DPH Gabriel Scally which proposed that, as the GMC did not want to take on the regulation of non-medical public health specialists, the role should be given to the Health Professions Council and made statutory for generalist specialists.^79^ The report concluded that formal training should be the single route to registration with ‘minimal exceptions’, thus quite explicitly suggesting ending the routine use of the UKPHR portfolio route.^80^ Initially, the response of the new Conservative/Liberal Democrat Coalition government was cool, indicating that they preferred to continue voluntary regulation. But following further lobbying including a strong recommendation from the House of Commons Health Committee, in 2012, the government eventually committed to statutory regulation by the Health and Care Professions Council.^81^ However, despite repeated restatements of this intention in the period 2012-2015, in late December 2015, the government surprised the field by announcing that it did ‘not consider that extending statutory regulation to this professional group is necessary’.^82^

This decision was welcome with relief by the UKPHR and many of its allies as a move to statutory regulation by the Health and Care Professions Council was seen as threatening several key developments in multidisciplinary public health. First, and most critically, it was unclear whether the Health and Care Professions Council would have wanted or been in a position to maintain the portfolio route to registration which had been such an important conduit for many public health professionals to achieve specialist registration. Second, it potentially spelt the end of defined specialists, as many in the public health medical world had continued to oppose this innovation, as reflected in the Scally report.^83^ Third, UKPHR would not have been viable purely as a regulator of practitioners and it was highly unlikely that the Health and Care Professions Council would also want to take on the voluntary registration of practitioners, as it did not run any other voluntary registration schemes or show any wish to work outside its statutory remit. Finally, the UKPHR was committed to and working towards revalidation for non-medical specialists to mirror the medical revalidation introduced by the GMC, and it was not clear whether the Health and Care Professions Council would or could do likewise.

## The profession of public health in the 2020s

By 2020, the Faculty membership was evenly split between medical members and those from other disciplines. In 2019, Maggie Rae was elected as the first non-medical president of the Faculty; Rae had also been one of the first cohort of non-medical DsPH appointed in 2001. For some years, non-medical training had been fully integrated into the medical public health registrar training programmes, so that there was open recruitment of candidates regardless of disciplinary background. This was a well-resourced five-year programme which often included a funded Master’s in Public Health in the first year, and for which the registrars received a standard NHS registrar’s salary. Employers including the four UK nations’ public health agencies, the NHS, and local authorities in England accepted GMC and UKPHR specialist registration equally as an essential criterion for appointment as a DPH or consultant in public health. The UKPHR was financially stable and accepted as a key public health agency with a seat and a voice at important inter-agency public health discussions and decision-making. Although some public health doctors continued to grumble about the UKPHR’s portfolio route, it had become accepted as a permanent and legitimate pathway to specialist registration. Indeed, at times of crisis such as the COVID-19 pandemic, the Department of Health and Social Care made additional funds available to speed the assessment of portfolios and expand specialist capacity.^84^

Despite this seemingly rosy picture for multidisciplinary public health, tensions and complexities remained concerning the relationship of medical and non-medical public health. Salaries, and terms and conditions, of non-medical specialists not only continued to be worse than those for public health doctors, but the inequalities have worsened over time. The new Labour government’s settlement of the NHS doctors’ long-running pay dispute in 2024 significantly increased public health doctors pay in comparison with non-medical colleagues. In addition, despite various representations, only medical public health specialists have been eligible for academic clinical fellowships and clinical excellence (now impact) awards, significantly enhancing their salaries.^85^ Similarly, in England, the divide between an increasing proportion of medical specialists in the NHS and central government agencies (which offered NHS pay and terms and conditions), and an increasing proportion of non-medical specialists in local authorities (which generally offered worse pay and terms and conditions) grew wider over time. Some public health doctors continued to question the equivalence of the new specialist registration by portfolio assessment with the training scheme, in particular, the failure (in their eyes) of an adequate requirement for communicable disease control/health protection experience in the portfolio route. The UKPHR as an organisation remains extremely small and vulnerable to policy change compared with the GMC. As registration with it was notionally voluntary, there was continuing anxiety in the field that some English local authorities might no longer respect the convention that specialist registration was an essential criterion for consultant appointments. The training, career development, and potential registration of non-medical professionals at intermediate levels between practitioner and specialist registration remained an unresolved and contested issue. Various attempts over the years to identify and support a senior practitioner status had been unsuccessful. Most public health practitioners continued to need to put together ‘do it yourself’ careers with a large element of self-funding and no clear career pathways. And voluntary practitioner registration with UKPHR remained the exception rather than the rule, with registration numbers apparently plateaued at between 500 and 600 out of the estimated 10,000 to 15,000 public health practitioners in the UK.^86^

## Conclusions

A range of factors came together in the late 1990s to facilitate the change from a profession synonymous with a medical specialty to a multidisciplinary profession. The emergence of the health promotion movement in the 1970s and its increasing professionalism and academic standing in the 1980s and 1990s were crucial. The increase in academic units, Master’s programmes, researchers, and practitioners educated to Master’s and Doctoral levels meant there was by the 1990s a cadre of increasing knowledgeable, skilled, and ambitious health promotion professionals in public health services. In the mid-1990s, they allied with the pre-existing group of public health academics in established university academic departments who had been marginalised by the Faculty. The failure of health education/promotion officers to achieve recognition as a profession in the 1990s, and the closeness of health promotion practice to public health practice, particularly in areas like collaborative working, community development, and tackling health inequalities meant that by 1995 there was a large cadre of relevant professionals agitating for recognition as multidisciplinary public health specialists. With the election of the New Labour government in 1997, committed to public health and sceptical of professional protectionism, the tipping point had been reached for the Faculty, whose executive (if not all members) realised that the momentum towards opening Faculty membership and senior NHS posts was now virtually unstoppable.

The context was now entirely different from the 1968-1972 debates about non-medical membership in formation of the Faculty; then there was not the cadre of senior non-medical service public health people demanding change. In that earlier period, all the senior non-medical people in the Society for Social Medicine were academics. There were other occupational groups in service public health, but they were so distinct (e.g. sanitary engineers, social workers, health visitors) that there was no cadre of non-medical professionals seeking equivalence with public health doctors; by the 1990s there was much more of a spectrum with a number of occupations with claims of (potential) equivalence (health promotion professionals, epidemiologists, other doctoral-level researchers). Moreover, in the 1960s and 1970s, there was no sense of non-hierarchical multidisciplinary working as emerged in health promotion in the 1980s.

In terms of the public health medicine professional project, medical resistance to multidisciplinary public health changed from straightforward opposition to non-medical specialist appointments to senior posts in the early to mid-1990s (which became politically unsustainable), to a more subtle effort from 2000 to ensure multidisciplinary public health remained enmeshed in a largely unquestioned web of medically dominated structures (i.e. training, examinations, registration, regulation). Resistance to non-medical specialists had morphed into resisting breaking down barriers between elite specialists and other lower-status practitioners in public health. And the advocates of multidisciplinary public health largely accepted the medical framing of the profession. Thus, training for non-medical specialists was incorporated into the medical specialist training programmes within medical deaneries, a traditional elite medical model with limited training numbers but a well-funded five-year training programme not available to other practitioners in public health. Looking internationally, however, countries have many different ways of training public health leaders; for example the USA has multidisciplinary public health leaders, but no similar medically modelled training programme, instead a range of career pathways primarily based on Master’s and Doctoral degrees in Public Health.^87^

There has also been a common assumption between UK public health doctors and the advocates of multidisciplinary public health that professional regulation and registration of non-medical specialists along lines similar to those of the GMC was essential, thus leading to the birth of UKPHR. The GMC retained a key role in overseeing the public health training curriculum, and there were several attempts to shift multidisciplinary regulation to the GMC. Resistance to UKPHR portfolio route to specialist registration was explicitly around concerns that this would be to a lesser professional standard and allow those not of elite calibre to register as specialists. UKPHR defined specialists were by definition not able to demonstrate specialist-level competency across all domains of public health practice, but some of them have gone on to be very well-regarded DsPH.

From 2013, the growing difference in employment conditions between mainly non-medical specialists employed in English local authorities versus mainly medical specialists in the NHS and national public health agencies has established a new and different medical/non-medical divide. This fragmentation has been exacerbated by many English local authorities employing non-medical ‘public health specialists’ at lower grades and salaries than consultants in public health, and who are not registered with UKPHR as specialists. Along with the lower salaries offered to DsPH and consultants in English local authorities, this would appear to substantiate the fears expressed from at least the 1990s onwards by public health doctors that they might be replaced by cheaper non-medical equivalents.

To date, these anxieties do not appear to have been shared by most of those writing about the rise of multidisciplinary public health, many of whom like Sim, Griffiths, and Wright were also active participants in developing public health as a multidisciplinary profession. They appear to share common assumptions that the way multidisciplinary public health has developed is appropriate; in particular, most commentators from the public health field have assumed that professional regulation and registration as close as possible to that developed by the GMC were good things that protect the public. For a supposedly evidence-based profession, however, it is notable that no actual evidence has been presented that this is the case.

There is now a substantial literature on the regulation of health professions, but mainly on clinical health professionals, with little specifically on the regulation of public health professionals.^88^ Generally, regulation is seen as a good thing and necessary, but there is limited evidence on the best approaches and the relative costs and benefits of different approaches. Despite most of the literature focusing on clinicians, there is limited evidence on the impact of the regulation of clinical health professionals on patient safety, and no evidence specifically on the impact of the regulation of public health professionals on the health and welfare of the population. In the public health literature advocating registration, the costs of the regulation are virtually never discussed. There is one brief mention in Sim and Griffiths indicating that initially some in the Department of Health were not convinced that regulation of non-medical public health specialists was necessary to protect the public health.^89^ Statutory regulation is usually portrayed by public health commentators as superior to voluntary regulation, and sometimes assumed to naturally follow in due course. There is a tendency in most accounts to take a somewhat celebratory approach to reporting progress with regulation, but not recognising the tensions and complexities outlined above. There appears no recognition that the multidisciplinary project is at all vulnerable and could potentially unravel. For example, neither the detailed account by Wright, Sim, and Ferguson in 2016 nor their subsequent paper in 2022 considers the existential threat to UKPHR’s existence c. 2013-2015 that might have led to its demise and thus potentially to a crisis in the multidisciplinary project.^90^

Most fundamentally, previous accounts have failed to critique the binary medical model of public health specialist training and registration (i.e. either you are an elite specialist or a lower-level practitioner), whereas public health practice is much more of a spectrum of expertise and experience where people do not necessarily fit easily into the binary divide shaped by the current model of specialist registration. The portfolio route has allowed a safety valve for some practitioners to demonstrate that through work-based learning and continued professional development they have achieved expert levels of knowledge and skill equivalent to that gained on the training scheme. But the exacting demands of the portfolio route as constituted have meant that only a small proportion of practitioners have gained specialist status through this route. Thus, the tensions around career development for practitioners and the challenging route to specialist status that have existed since the 1990s have continued. Whether it was strictly necessary or not to protect the public, introducing regulation of non-medical public health specialists closely modelled on that of the GMC was crucial for winning Faculty and wider public health medical support for opening senior public health roles to those from backgrounds other than medicine. The failure of health promotion to achieve specialist status due to the lack of Department of Health and employer support, will not have been lost on the advocates of multidisciplinary public health in the 1990s, a number of whom came from health promotion backgrounds.

Finally, the question for the future is whether this binary model will be sustained, given the financial and other pressures public health employers face and the constraints this model places on public health workforce development. The history of the last fifty years suggests that the profession (particularly as represented by the Faculty) will continue to seek to maintain the professional project and the boundary between elite public health specialists and lower-status public health practitioners. Inequalities between medical and non-medical specialists will continue as the factors determining these inequalities are too complex and difficult to easily fix. There are likely to be some marginal improvements in the pathways from practitioner to specialist status, along the lines of recent enhanced regional support programmes for those seeking specialist registration by portfolio assessment.^91^ The wider professional development of public health practitioners is likely to continue to be the subject of debate, and further workforce plans, as it has been for decades, but in the current financial climate, it is difficult to envisage the substantial new resource that would be required to implement any significant improvement in practitioner development.^92^ The widening divide between public health doctors employed in the NHS and national agencies and non-medical specialists employed in English local authorities is likely to continue due to increasing salary and terms and conditions differences. There continues to be a risk that some English local authorities may cease to honour the non-statutory agreement to require consultants in public health to be on the specialist register and it is even more likely that due to financial pressures, more will substitute cheaper non-registered ‘specialists in public health’ for more expensive consultants. However, if there is one overarching lesson that can be taken from the history of the UK profession of public health over the last fifty years, it is that the profession is likely to be buffeted by the unanticipated consequences of wider policy developments. The local government and NHS reforms of the early 1970s, the introduction of the NHS internal market in the 1990s, and the transfer of the local public health function in England from the NHS to local authorities in 2013 all had profound and unanticipated consequences for the profession of public health. Thus, the profession in 2025 is very different from that of 1970, despite the underlying continuity in the public health professional project that privileges elite recruitment, extensive training, expert knowledge, professional registration, and regulation.

## Footnotes

1

Health Education England, A Capacity Review: Public Health Specialists in 2022 (London: Health Education England, 2022).

2 The only other UK medical specialty which allows senior roles to candidates from other disciplines is pathology, where clinical scientists can be appointed as fellows of the Royal College of Pathologists and consultant scientists within the NHS. However, non-medical scientists were included in the Royal College of Pathologists from its inception in 1962, so there was not a similar process of opening what had previously been an exclusively medical profession.

3

Jane Lewis , What Price Community Medicine? The Philosophy, Practice and Politics of Public Health Since 1919 (Brighton: Wheatsheaf, 1986); Jane Lewis , ‘The changing fortunes of community medicine’, Public Health, 100, 1 (1986), 3–10 3749436 10.1016/s0033-3506(86)80079-8; Jane Lewis , ‘From public health to community medicine’, in Stephen Farrow (ed.), The Public Health Challenge (London: Hutchinson Education, 1987), 87–99 ; Jane Lewis , ‘The public’s health: philosophy and practice in Britain in the twentieth century’, in Elizabeth Fee and Roy Acheson (eds), A History of Education in Public Health (Oxford: Oxford University Press, 1991), 195–229 .

4 Lewis, ‘The public’s health’, *op. cit.* (note 3), 197.

5

Martin Gorsky , ‘Local leadership in public health: the role of the medical officer of health in Britain, 1872–1974’, Journal of Epidemiology and Community Health, 61 (2007), 468–472 17496253 10.1136/jech.2006.046326PMC2465709; Martin Gorsky , ‘Public health in interwar England and Wales: did it fail?’, Dynamis, 28 (2008), 175–198 19230339 10.4321/s0211-95362008000100008PMC2647660; Martin Gorsky , Karen Lock and Sue Hogarth , ‘Public health and English local government: historical perspectives on the impact of “returning home”’, Journal of Public Health, 36, 4 (2014), 546–551 24472776 10.1093/pubmed/fdt131PMC4245898.

6

Virginia Berridge , Daphne Christie and Tilli Tansey (eds.), Public Health in the 1980s and 1990s: Decline and Rise? (London: The Wellcome Trust Centre for the History of Medicine at UCL, 2006); Donald Acheson , Public Health in England (London: HMSO, 1988).

7

Michael Warren , ‘The creation of the Faculty of Community Medicine (now the Faculty of Public Health Medicine) of the Royal Colleges of Physicians of the United Kingdom’, Journal of Public Health Medicine, 19 (1997), 93–105 9138225 10.1093/oxfordjournals.pubmed.a024596; Michael Warren , The Origins of the Faculty of Public Health Medicine (London: Faculty of Public Health Medicine, 2000); Berridge, Christie, and Tansey, *op. cit.* (note 6).

8 As Berridge has pointed out, public health as a wider movement and academically was multidisciplinary throughout this period, but it was public health as an occupation and a profession which was narrowly medical and obsessed with status: Virginia Berridge , ‘Multidisciplinary public health: What sort of victory?’, Public Health, 121 (2007), 404–817451764 10.1016/j.puhe.2007.02.004.

9 Berridge, Christie & Tansey *op. cit.* (note 6)*;* David Evans and Teri Knight , ‘There Was No Plan!’ The Origins and Development of Multidisciplinary Public Health in the UK (Bristol: University of the West of England, 2006); Teri Knight and David Evans , ‘History of multidisciplinary public health mini-symposium’, Public Health, 121 (2007) 401–317459435 10.1016/j.puhe.2007.03.011.

10

Jenny Wright , Fiona Sim and Katie Ferguson , Multidisciplinary Public Health (Bristol: Policy Press, 2016). The authors are public health professionals, and Sim and Wright were both active in the development of multidisciplinary public health in the 1990s and early 2000s.

11

David Evans , ‘“Taking public health out of the ghetto”: the policy and practice of multidisciplinary public health in the United Kingdom’, Social Science & Medicine, 57 (2003), 959–6712878097 10.1016/s0277-9536(02)00473-2; Eliot Freidson , Profession of Medicine: A Study of the Sociology of Applied Knowledge (New York: Dodd, Mead & Co, 1970); Magali Larson , The Rise of Professionalism: A Sociological Analysis (Berkeley: University of California Press, 1977).

12

Susan Nancarrow and Alan Borthwick , The Allied Health Professions: A Sociological Perspective (Bristol: Policy Press, 2021); Gerry Larkin , ‘The regulation of the professions allied to medicine’ in Judith Allsop and Mike Saks (eds.), Regulating the Health Professions (London: Sage, 2002).

13

Sola Decker and Ron Iphofen , ‘Developing the profession of radiography: making use of oral history’, Radiography, 11 (2005), 262–71; Leo McCann , Edward Granter , Paula Hyde and John Hassard , ‘Still blue-collar after all these years: an ethnography of the professionalization of emergency ambulance work’, Journal of Management Studies, 50 (2013), 750–76; Jenny Bangham , ‘Making the ‘genetic counsellor’ in the UK, 1980–1995’, Medical Humanities, 49 (2023), 248–5937068944 10.1136/medhum-2022-012472PMC10359581.

14
*Professions Supplementary to Medicine Act 1960.*

15 Evans, *op. cit.* (note 11), 963.

16 Gorsky, Lock and Hogarth, *op. cit.* (note 5).

17 Peter Duncan has written an important history of the rise and fall of health promotion as a specialism, which tangentially mentions the links with the rise of multidisciplinary public health but does not explore this aspect in detail: Peter Duncan , ‘Failing to professionalise, struggling to specialise: the rise and fall of health promotion as a putative specialism in England, 1980–2000’, Medical History, 57, 3 (2013), 377–396 24069885 10.1017/mdh.2013.19PMC3865947.

18

Roy Acheson , ‘The British Diploma in Public Health: heyday and decline’, in Elizabeth Fee and Roy Acheson (eds), A History of Education in Public Health (Oxford: Oxford University Press, 1991), 272–313 .

19
*Ibid.*; Walter Holland , ‘Public health coming home’, Journal of Public Health, 37, 2 (2015), 362–325724611 10.1093/pubmed/fdv024.

20

Walter Holland and Susie Stewart , Public Health: The Vision and the Challenge (London: Nuffield Trust, 1998), 111 .

21 Warren, ‘The creation of the Faculty’, *op. cit.* (note 7).

22 Holland and Stewart, *op. cit.* (note 19), 114; Berridge, Christie, and Tansey, *op. cit.* (note 6), 40, 54.

23 Frederick Seebohm, *Report of the Committee on Local Authority and Allied Personal Social Services* (Seebohm Report) Cm 3703 (London: HMSO, 1968); Royal Commission, *Report on Local Government in England 1966–1969* Cmnd 4040 (London: HMSO, 1969); *Local Authority Social Services Act 1970.*

24 Warren, ‘The creation of the Faculty’, *op. cit.*; Warren, *The Origins of the Faculty, op. cit.* (note 7).

25 MOsH themselves were generally in favour of the transition to community medicine as they saw it as giving them an important new role in the NHS and raising their status in the medical world: Lewis, ‘The changing fortunes of community medicine’, *op. cit.* (note 3), 5.

26 Royal Commission, *Report on Medical Education 1965–1968*, Cmnd 3569 (Todd Report) (London: HMSO, 1968).

27 Warren, ‘The creation of the Faculty’, *op. cit.* (note 7), 93.

28
*Ibid.*, 97.

29 These appointments followed a Department of Health and Social Security commissioned review, which saw community physicians primarily as medical administrators: Department of Health and Social Security, *Report of the Working Party on Medical Administration (Hunter Report)* (London: HMSO, 1972).

30 Holland and Stewart, *op. cit.* (note 19), 126-43; Lewis, *What Price Community Medicine, op. cit.* (note 3), 125–59*;* Berridge, Christie, and Tansey, *op. cit.* (note 6), 11–29.

31 Evans and Knight, *op. cit.* (note 9), 27.

32 Department of Health and Social Security, *NHS Management Inquiry Report* (Griffiths Report) (London: Department of Health and Social Security, 1983).

33 Lewis, ‘The changing fortunes of community medicine’, *op. cit.* (note 3), 5; Holland and Stewart, *op. cit.* (note 19), 129; Acheson, *op. cit.* (note 6), 8.

34 Unit for the Study of Health Policy, *Rethinking Community Medicine: A Report from a Study Group* (London: Unit for the Study of Health Policy, 1979)

35
*Ibid.*, 99.

36

Linda Ewles , ‘Paddling upstream for 50 years: the role of health education officers’, Health Education Journal 52 (1993), 172–81.

37
*Ibid.*

38

Marc Lalonde , A New Perspective on the Health of Canadians (Ottawa: Government of Canada, 1974); WHO Europe, *Ottawa Charter for Health Promotion* (Geneva: WHO, 1986); WHO, *Global Strategy for Health for All by the Year 2000* (Geneva: WHO, 1981).

39

John Ashton and Howard Seymour , The New Public Health (Milton Keynes: Open University Press Books, 1988).

40 Acheson, *op. cit.* (note 6). Acheson was Chief Medical Officer for England from 1984 to 1991.

41
*Ibid.*, 6.

42
*Ibid.*, 56.

43
*Ibid.*, 10–11.

44
*Ibid*, 11–12.

45 Berridge, Christie, and Tansey, *op. cit.* (note 6), 19–25.

46 Department of Health, *Working for Patients*, Cm 555 (London: HMSO, 1989).

47 Ewles, *op. cit.* (note 36), 176.

48 Berridge, Christie, and Tansey, *op. cit.* (note 6), 50–54.

49 Berridge, Christie, and Tansey, *op. cit.* (note 6), 57.

50

Lillian Somervaille and Rod Griffiths , The Training and Development Needs of Public Health Professionals (Birmingham: University of Birmingham, 1995), 1.

51
*Ibid.*, 6.

52
*Ibid.*, 7.

53 Wright, Sim, and Ferguson, *op. cit.* (note 10), 41.

54
*Ibid.*, 42–4; Berridge, Christie, and Tansey, *op. cit.* (note 6), 55–58.

55 Berridge, Christie, and Tansey, *op. cit.* (note 6), 57; Evans and Knight, *op. cit.* (note 9), 64.

56 Duncan, *op. cit.* (note 17), 388–9.

57 Ewles, *op. cit.* (note 36), 177.

58 Duncan, *op. cit.* (note 17), 391.

59 The Society of Health Education and Health Promotion Specialists limped on into the early 2000s, but then ceased to operate.

60 Chief Medical Officer for England 1991–1998.

61

Suszy Lessof , Carol Dumelow and Klim McPherson , Feasibility Study of the Case for National Standards for Specialist Practice in Public Health (London: London School of Hygiene and Tropical Medicine, 1999).

62 Kenneth Calman, *Chief Medical Officer’s Project to Strengthen the Public Health Function in England: A Report of Emerging Findings* (London, Department of Health, 1998). The final version of this report was issued by Calman’s successor, Liam Donaldson: Department *of Health*, *The Report of the Chief Medical Officer’s Project to Strengthen the Public Health Function* (London: Department *of Health, 2001).* Neither Calman nor Donaldson appears to have written or spoken publicly in support of multidisciplinary public health at the time other than in this report, but Donaldson later wrote of his ‘strong support’ when he became Chief Medical Officer in his Foreword to Wright, Sim, and Ferguson, *op cit.* (note 10), vii. Calman demonstrated his continued support by agreeing to be the first chair of the Advisory Group for the UK Voluntary Register for Public Health Specialists (see below).

63 Chief Medical Officer for England 1998–2010.

64 Wright, Sim, and Ferguson, *op. cit.* (note 10), 48.

65

Sian Griffiths , June Crown and Jim McEwen , ‘The role of the Faculty of Public Health (Medicine) in developing a multidisciplinary public health profession in the UK’, Public Health, 121 (2007), 420–517462683 10.1016/j.puhe.2007.02.021.

66 Secretary of State for Health, *Saving Lives: Our Healthier Nation*, Cm 4386 (London: Stationery Office, 1999), 136.

67

Paul Pilkington , Sally Dowling , Geoffrey Barnes , Tessa Lindfield , and Alison Pritchard , ‘Trainees’ experiences of multidisciplinary public health training schemes in England’, Public Health, 121 (2007), 432–717459433 10.1016/j.puhe.2007.02.007.

68

Fiona Sim , Jenny Wright , and Katie Ferguson , ‘Creating a robust multidisciplinary public health workforce – almost there?’, Journal of Public Health 44 (2022), i40–8, i4336465048 10.1093/pubmed/fdac090.

69

Fiona Sim and Jenny Griffiths , ‘Origin and development of the UK Voluntary Register for Public Health Specialists’, Public Health, 121 (2007), 443–448, 44417433388 10.1016/j.puhe.2007.02.022.

70 For example, Klim McPherson , Sarah Taylor , Edward Coyle , ‘For and against: public health does not need to be led by doctors’, British Medical Journal 322 (2001), 1593–611431305 10.1136/bmj.322.7302.1593PMC1120630.

71 Alan Milburn, *A Healthier Nation and a Healthier Economy: The Contribution of a Modern NHS* (LSE Annual Lecture, 8 March 2000).

72 Wright, Sim, and Ferguson, *op. cit.* (note 10), 60.

73 Sim and Griffiths, *op. cit.* (note 69).

74 Evans and Knight, *op. cit.* (note 9), 41.

75 Wright, Sim, and Ferguson, *op. cit.* (note 10), 84.

76 The UKPHR specialist registration by portfolio assessment route is intended to be comparable to the GMC Certificate of Eligibility for Specialist Registration (CESR) route for doctors, although the numbers are much smaller for the latter scheme, https://ukphr.org/specialist-registration-by-portfolio-assessment/ (accessed 23 December 2024).

77 Secretary of State for Health, *Health Lives, Healthy People: Our Strategy for Public Health in England* (London: HMSO, 2010).

78 For example, ‘senior public health specialist posts’ advertised by Bristol City Council in 2019 and Somerset County Council in 2021, neither of which required specialist registration.

79 Department of Health, *Review of the Regulation of Public Health Professionals* (London: Department of Health, 2010).

80
*Ibid.*, 40.

81 House of Commons Health Committee, *Public Health* (London: Stationery Office, 2011); Secretary of State for Health, *Government Response to the House of Commons Health Committee Report on Public Health* (London: Stationery Office, 2012). The Health Professions Council’s name was changed to the Health and Care Professions Council when responsibility for the regulation of social workers was transferred to it in 2012.

82 Parliamentary Under-Secretary of State for Health, ‘Regulation of Health and Social Care Professionals,’ Hansard 603 (2015), debated on Thursday 17 December 2015. As late as 2023, some public health doctors in the Faculty were still suggesting that the GMC should be given the role of statutory regulation of non-medical public health specialists.

83 Department of Health, 2010 *op. cit.* (note 79), 40.

84 UKPHR, *Increasing the Supply of Public Health Consultants with Additional Portfolio Assessor Resource* (7 October 2020), https://ukphr.org/news/increasing-the-supply-of-public-health-consultants-with-additional-portfolio-assessor-resource/ (accessed 23 December 2024)

85 Faculty of Public Health, *National Clinical Impact Awards (NCIA) 2025*, https://www.fph.org.uk/professional-development/workforce/clinical-impact-awards/#:~:text=You%20can%20apply%20for%20a,with%20a%20Licence%20to%20Practise (accessed 23 December 2024).

86 UKPHR, *Annual Report and Financial Statements 2023/23* (Birmingham: UKPHR, 2024). There is no agreed definition of public health practitioner, nor definitive census of numbers, but the Centre for Workforce Intelligence estimated up to 10,000 public health practitioners plus around 5,000 public health managers, scientists, and intelligence and knowledge professionals who might also be included: Centre for Workforce Intelligence, *Mapping the Core Public Health Workforce* (London: CfWI, 2014).

87 Institute of Medicine, *Who Will Keep the Public Healthy? Educating Public Health Professionals for the 21st Century* (Washington, DC: The National Academies Press, 2003).

88

Kathleen Leslie et al., ‘Design, delivery and effectiveness of health practitioner regulation systems: an integrative review’, Human Resources for Health, 21 (2023), 72 37667368 10.1186/s12960-023-00848-yPMC10478314.

89 Sim and Griffiths, *op. cit.* (note 69), 446.

90 Wright, Sim, and Ferguson, *op. cit.* (note 10); Sim, Wright, and Ferguson*, op. cit.* (note 68).

91
*UKPHR Specialist Registration by Portfolio Assessment (SRbPA): An Alternative Pathway to Public Health Specialist Recognition*, https://london.hee.nhs.uk/specialty-schools/welcome-school-public-health/ukphr-specialist-registration (accessed 27 December 2024).

92 The most significant recent development in practitioner training has been the introduction of public health practitioner degree apprenticeships, but this is an entry-level qualification, not a stepping stone from practitioner to specialist status: *Public Health Practitioner (integrated degree) Apprenticeship*, https://ukphr.org/2-public-health-practitioner-integrated-degree-apprenticeship/ (accessed 27 December 2024).

